# Experimental Evidence of Large Amplitude pH Mediated Autonomous Chemomechanical Oscillation

**DOI:** 10.3390/polym9110554

**Published:** 2017-10-25

**Authors:** Xin Yang, Yi Zhou, Lin Ji, Yanhui Ding, Jianquan Wang, Xin Liang

**Affiliations:** 1 Department of Chemistry, Capital Normal University, Beijing 100048, China; sophieyangyifan@163.com (X.Y.); linus.zhou@gmail.com (Y.Z.); dingyh_0411@163.com (Y.D.); 2 School of Materials Science and Engineering, Beijing Institute of Technology, Beijing 100081, China; 3 Xi’an Satellite Control Center, Xi’an 710043, China; liangxin20072007@126.com

**Keywords:** autonomous chemomechanical system, pH responsive hydrogel

## Abstract

Large amplitude autonomous chemomechanical oscillations were observed in a coupled system consisting of a porous pH-responsive hydrogel and a bromate-sulfite-manganese (II) pH oscillatory reaction. The porous structure effectively improves the chemomechanical response speed, and the negative feedback species of the bulk oscillation Mn^2+^ takes part in the coupling by forming complex and physical crosslinks with the responsive group in the gel. It strengthens the porous gel by forming additional networks, which may contribute to sustaining the long-lasting chemomechanical oscillation. Additionally, the interaction between Mn^2+^ and the hydrogel alters the period of the oscillatory reaction due to its binding competition with H^+^, the positive feedback species.

## 1. Introduction

Stimuli-responsive hydrogels have recently been coupled with oscillatory chemical reactions to produce autonomous chemomechanical materials, which have wide potential applications in many areas [1,2,3,4,5,6,7] such as drug release [1], homeostatic materials [4], and biomimetic actuators [5,6,7]. Autonomous chemomechanical systems can generally be classified into two categories. In the first type, the mechanical oscillations of the gel are merely slaved to the chemical oscillation kinetics. The gel is immersed in oscillatory reaction components [2,8,9,10,11] or the oscillations can take place inside the gel when introducing appropriate reactants into the polymer network [5,6,12,13,14] or when the reactant species act as an active cross-linker of the gel [15]. In the second type, instabilities are generated by the interactions between the gel and non-oscillatory reaction, which can be further classified into two scenarios: “long-range activation” and “synergetic chemomechanical oscillation”. In the case of long-range activation [16,17], differential diffusion contributes to the spatiotemporal oscillations. In synergetic chemomechanical [4,18,19,20,21,22,23,24,25] oscillations realized in spatial bistable systems [18,19,20,23,24] or enzymatic reactions realized in reactive porous membranes [21,22], gel size change exerts feedback on the reaction so that an oscillation can be induced in non-oscillatory bulk reactions.

Although pH-responsive gels are easy to obtain, and there are many pH oscillators available [26], the realization of pH-mediated autonomous chemomechanical systems is not so expected. Yoshida et al. had reported a self-oscillating gel by coupling p(*N*-Isopropylacrylamide-*co*-acrylic acid) with a pH oscillatory reaction [8]. pH responsive hydrogel polymethacrylic acid was also realized to oscillate in a bromate-sulfite-ferrocyanide pH oscillation reaction [9]. Additionally, the gels employed in the reported systems are often small. One of the constraints is the dependence of the rate of volume change of the gel on the mass transport of the solvent [2], whereby the rate of volume change is inversely proportional to the square of the linear dimension of the hydrogel [27]. Accordingly, large pH-responsive gels do not have enough time to change size on the time scale of the pH oscillation period, which is often tens of minutes [10,11]. Incorporating pores into hydrogels [10,11,28,29,30] to facilitate the migration of water through enlarging the surface area is often employed to improve the hydrogel response speed. Additionally, the oscillatory reaction constitutes an environment with high ion strength. Obtaining large amplitude responsive size changes is a demanding requirement for pH sensitive hydrogel, which is a polyelectrolyte in nature. Moreover, reversible volume changes may induce high strains on the gel network, and such effects are more significant if the gel is large. Chemical cross-linking in pH-responsive gels inevitably introduces spatial heterogeneity in the cross-link density, leading to local anisotropic expansion and contraction. Such an expansion can instigate network fracture, thus lowering the mechanical strength of the gel [2,31,32]. In the present study, a porous pH-sensitive hydrogel with a special porogen alginate is synthesized to accelerate the diffusion inside the gel. The bromate-sulfite-manganese (II) (BSM) pH oscillatory reaction [33], which provides large amplitude periodical pH changes, was employed as the driving force. In this reaction, Mn^2+^ (or MnO_4_^−^) acts as the negative feedback species that consumes H^+^, and the reaction of BrO^3−^-SO_3_^2^^−^ in an acidic environment produces H^+^ in an autocatalytic manner. The hydrogel interacts with the oscillation not only through H^+^, but also through the negative feedback species Mn^2+^. The special interaction between Mn^2+^ and the hydrogel strengthens the gel by forming complex and physical crosslinks and weakens the salt effect. Although the chemomechanical instability of this system still originates from the original oscillatory kinetics, the oscillation ingredient also contributes to strengthen the gel and bring about the large amplitude chemomechanical oscillation.

## 2. Materials and Methods

### 2.1. Hydrogel Synthesis

Hydrogel poly(acrylic acid-*co*-acrylamide) (p(AA-*co*-AM)) was synthesized by free-radical solution polymerization. The monomer acrylamide (Amethyst Chemicals, Beijing, China), acrylic acid (Alfa Aesar, Ward Hill, MA, USA), (monomer ratio = 1:1 (mass ratio), concentration of monomers in pregel solution: 10 wt %), cross-linker *N*,*N*′-methylenebis(acrylamide) (0.4 wt % of total monomer, Sinopharm Chemical Reagent, Beijing, China), initiator ammonium persulfate (2 wt % of total monomer, Sinopharm Chemical Reagent, Beijing, China), and porogen sodium alginate (SA; viscosity ≥ 0.02 Pa∙s, 29.3 wt % of total monomer, Sinopharm Chemical Reagent, Beijing, China) were dissolved in 0.6 M NaCl solution in a glass beaker. The salt solution was used to instigate a coil-to-globule transition of the SA chains so that a porous structure is obtained after the SA chains are washed out. Then, the glass beaker was sealed and transferred to a thermostatic water bath at 65 °C for 4 h. Finally, the mixture was cooled to room temperature. Hydrogels were immersed in deionized water for seven days, with regular water replacement to eliminate impurities. Before testing, all the fully-swollen samples were immersed in buffer solutions of pH 6.8 for 12 h and stained with methylene blue. Samples prepared in this way are referred to as the porous hydrogels. For comparison, non-porous hydrogels were also synthesized by similar processes without adding SA and NaCl. The morphology of the porous and non-porous hydrogels was observed by scanning electron microscopy (SEM; SU8010, Hitachi, Tokyo, Japan). The element component of gel soaked in oscillatory reaction is tested by energy dispersive spectrometer (EDS; SU8010, Hitachi, Tokyo, Japan). The gel component was characterized by Fourier transform infrared (FT-IR; Bruker Tensor 27, Karlsruhe, Germany) spectroscopy. The bulk gel synthesized in the beaker was cut into cubes with a razor blade.

### 2.2. pH Oscillation Setting

Oscillation experiments were performed in a water-jacketed glass continuous stirred tank reactor (CSTR, 24 mL). The reaction temperature was maintained to 45 ± 0.l °C by circulating water between the water jacket and the thermostatic water bath (PolyScience 9006, Niles, IL, USA). Four stock solutions of BSM reactant, containing SO_3_^2−^, H^+^, Mn^2+^, and BrO_3_^−^, respectively, were separately introduced into the reactor by a peristaltic pump (Waston Marlow-205S, Cornwall, UK). The volume of the mixture in the reactor was maintained by continuous aspiration of the solution from the top of the reactor. The solution mixture in the CSTR was stirred magnetically by a Teflon-coated magnetic stirrer bar. Sufficient time was maintained at each flow rate to obtain stable oscillatory states. The pH was monitored using a combined glass electrode connected to a data acquisition system (CONSORT D230, Turnhout, Belgium).

### 2.3. Hydrogel pH-Responsiveness Test

The gel responsiveness to the pH oscillator was tested using both direct and indirect methods. In the direct method, the hydrogel was coupled with the BSM oscillation by suspending the sample on a small plastic plate that was immersed in the CSTR solution. The volume changes of the gel were monitored by a video camera, and the corresponding volume (length) change was determined from the images. In the indirect method, the sample was alternately immersed in buffer solutions of pH 1.7 and 6.8 for 8 min, with its weight being recorded every 2 min. The ionic strength of the buffer solutions was adjusted to that of the BSM system [33], which is calculated with the input concentration.

## 3. Results and Discussion

### 3.1. Coupled Chemomechanical Oscillations

**Figure 1:**
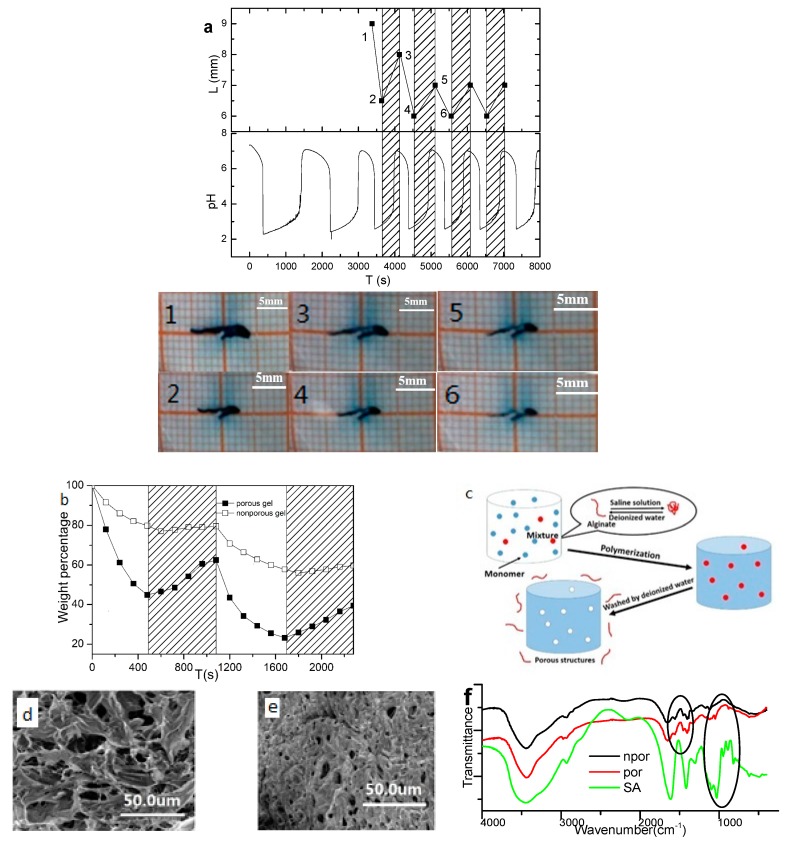
(**a**) Variations in the length of the porous gel and pH of the BSM system as a function of time. Snapshots were taken at the time points noted in the time sequence graph. The BSM oscillation parameters are [NaBrO_3_]_0_ = 90 mM, [Na_2_SO_3_]_0_ = 118 mM, [MnSO_4_]_0_ = 9 mM, [HClO_4_]_0_ = 16.5 mM, *T* = 45 °C, *k*_0_ = 2.945 × 10^−3^·s^−1^. (**b**) Comparison of the responsive swelling of the porous and non-porous gels determined in the offline study; the ionic strength of the buffer solution was adjusted to 0.5 M. The unshaded areas of the graph represent instances when the pH is 1.7, whereas the shaded areas of the graph represent instances when the pH is 6.8. (**c**) Schematic of the formation of the porous (gel) structure upon addition of SA to NaCl solution. SEM images of the (**d**) porous and (**e**) non-porous gels. FT-IR spectrum of porous, nonporous hydrogel (**f**).

As observed in the snapshot (Figure 1a) and the video (Supplementary materials), the porous p(AA-*co*-AM) hydrogel showed sustained reversible volume (length) changes with the pH changes of the BSM oscillation [33]. The BSM system provides a large amplitude pH change, whose period is long and its duration in the high pH parts are comparative to that in the low pH parts. The Mn^2+^ plays a clear and independent role of negative feedback in the mechanism. Here, the oscillation parameter setting was chosen under the principle that the oscillation is stable and the period is long. The measured length change was maintained at more than 10% for over 1 h, which is significant for the macroscopic chemomechanical energy transition. The contribution of porous acceleration is tested by control experiments of nonporous gels. Naked eye or charge-coupled devices (CCD) cannot recognize the volume change of the non-porous gel. Thus, the difference between the responses of the porous and non-porous gels was compared with an indirect test, whereby the weight of the gels was measured after the gels were soaked in buffer solutions. The periodic response of the porous hydrogel was considerably more obvious than that of the non-porous hydrogel (Figure 1b). In another quantitative test, the porous gel deswelled to 40% of its original weight in 8 min, whereas the non-porous gel took 32 min to achieve the same result. Therefore, the non-porous gel could not respond within the time scale of the oscillatory period to produce visible volume changes. The accelerated response rate in the porous gel was attributed to the porous structure formed upon contact of SA with NaCl solution. SA is a natural polyelectrolyte that undergoes a coil-to-globule transition when in contact with the NaCl solution to generate a porous gel structure (Figure 1c). The SEM images of the porous and non-porous gels in Figure 1d, e clearly demonstrate the pore-forming effect. Owing to the chain transfer reactions, part of the SA could graft to the copolymer [34,35]. FT-IR spectroscopy results (Figure 1f) of the porous gels have some very weak absorptions at 880 cm^−1^ and 1050 cm^−1^, which suggests the presence of residual SA. SA is proposed to graft to the hydrogel through the macroradicals obtained by abstracting hydrogen from the hydroxyl groups in SA by the sulfate anion radical (from the initiator [36,37]).

However, reversible, large amplitude chemomechanical oscillation is not realized by simply increasing the gel volume change rate. Control experiments with porous gels obtained by other methods, such as a gas blowing (or foaming) techniques or using silica particles as a porogen, showed no evidence of autonomous gel volume oscillation. Moreover, some samples do not swell in the indirect test at all. This is because the fragile nature of the gel network is accentuated since there are more walls inside the gel. Note that the ionic strength (~0.5 M) of the current chemomechanical system is considerably higher than that for other pH-responsive gels [38,39,40]. The good salt resistance displayed by the porous p(AA-*co*-AM) hydrogel here may be related to the special interaction of Mn^2+^ with the gel, which will be further discussed in later sections.

### 3.2. Mn^2+^ Coupled to the Hydrogel

**Figure 2:**
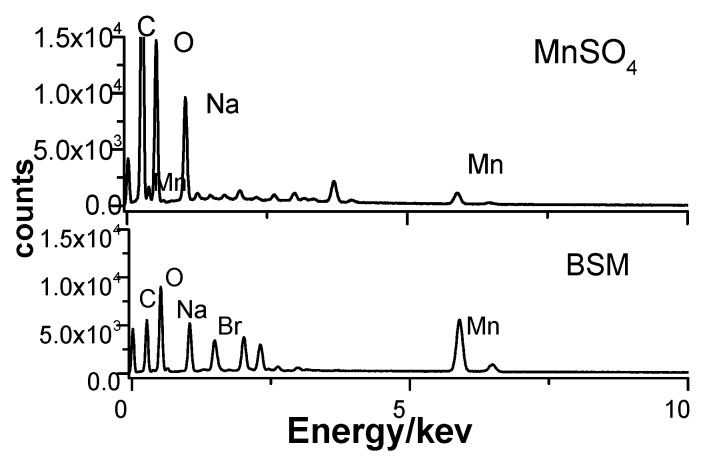
Energy dispersive spectrometry of the porous p(AA-*co*-AM) hydrogel taken from the 9 mM MnSO_4_ and BSM (with the same parameter as Figure 1a) solution.

Instead of being brittle and weak, the porous p(AA-*co*-AM) hydrogel appears to be even stronger when soaked in the oscillatory solution. It is speculated to be an effect of the Mn^2+^ involvement, which will interact with carboxyl groups, as well as the porogen SA. To identify the presence of Mn^2+^ in the hydrogel, energy dispersive spectrometry tests were performed for porous gels soaked in MnSO_4_ solution and BSM solution after they were washed with distilled water. EDS results in Figure 2 confirm the presence of Mn^2+^ in the gel, and the local Mn^2+^ content in the gel soaked in BSM solution is much higher than that in the MnSO_4_ solution. This might be because BSM is an open system with continuous input.

**Figure 3:**
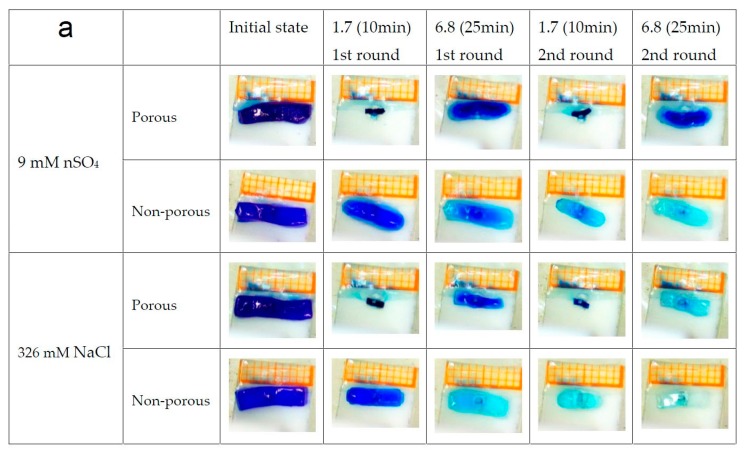

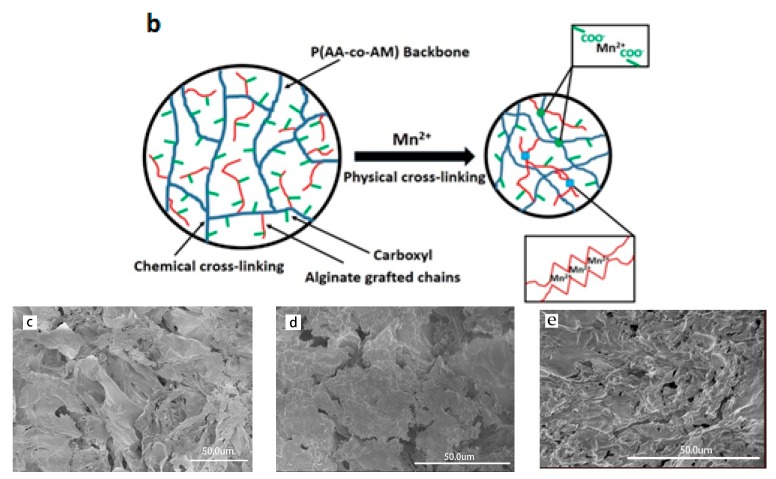
(**a**) Comparison of the swelling/deswelling behaviors of the porous and non-porous gels immersed in 9 mM MnSO_4_ and 326 mM NaCl of buffer solution with different pH. *T* = room temperature, initial pH: 6.8 (**b**) Schematic of the physical cross-linking between the porous gel and divalent metal ions. An additional network is formed upon interaction of Mn^2+^ with SA through an “egg carton” conformation and with carboxyl groups on AA through a complex conformation. SEM images of the (**c**) porous gel in MnSO_4_ solution, (**d**) non-porous gels soaked in MnSO_4_ solution, and (**e**) porous gel in NaCl solution.

The responsive performance of the porous (with SA) and non-porous (SA-free) gels were compared by alternatively immersing the gel in the buffer solutions of pH 1.7 and 6.8 with the addition of MnSO_4_ and NaCl, respectively. The ionic strength of the buffer solution was adjusted to be the same. The concentration of MnSO_4_ and NaCl in the solution are the same as that in the BSM system. Following the duration of low and high pH part in the BSM oscillation, the gels are first immersed in the pH 1.7 solution for 10 min, and then cleaned to put into the pH 6.8 solution for 25 min. This cycle is then repeated. It is obvious from the snapshots in Figure 3a that the nonporous gel shows only limited volume change due to the limited response speed, while the porous gels exhibit a definite reversible size change. Comparing the last snapshot in each group, the color of the gel fades rapidly, except for the porous gel in the Mn^2+^ solution. This indicates that there are some additional networks formed inside the gel with the aid of Mn^2+^ ions in solution, which helps to hold the dye molecules in the gel.

Previous investigations had reported the formation of complex of Mn^2+^ with polyelectrolytes, like ployacrylic acid [41,42]. Additionally, SA is known to possess a strong gelation capability in the presence of divalent cations [43,44]. The regular intramolecular configuration of SA facilitates the formation of an “egg carton” shaped physical cross-linked conformation with divalent cations (Figure 3b). Therefore, Mn^2+^ will lead to additional gel-oscillation interactions, especially with the presence of SA in the gel. These may finally form additional networks inside the hydrogel. Further experiments with SA and divalent cations show the binding of Mn^2+^ on SA in solution is quite limited compared with Ca^2+^ or Cu^2+^. In the hydrogel, this ability may be enhanced since SA molecules have low mobility when grafted on the polymer chain. This moderate strength of binding provides an appropriate physical crosslink network that strengthens the hydrogel network without destroying its responsivity. The color difference between the non-porous gel in Mn^2+^ and Na^+^ solutions suggests that the complex formation between Mn^2+^ and the gel can also form some networks inside the gel to prevent the dye molecules from leaving. However, probably due to the irregular distribution of AA on the gel chain, the strength of this latter network appears to be weaker compared to that with SA. From SEM images of the porous and nonporous gels soaked in the MnSO_4_ and NaCl solution (Figure 3c–e) we can see that there are still three-dimensional network structures in the porous gel sample after it was soaked in MnSO_4_ solution. For the nonporous gel or the porous gels soaked in NaCl solution, network structures are not clear. This suggests that there are complexation- and crosslinking-induced network structures in the presence of Mn^2+^. These interactions between Mn^2+^ and the gel suggests that Mn^2+^ in the solution influences the gel mainly by forming complex and physical cross-links, thus reducing electrostatic screening effects. Considering that Mn^2+^ contributes significantly to the overall ionic strength in the solution, this should be the main factor that improves the salt resistance of the gels.

**Figure 4:**
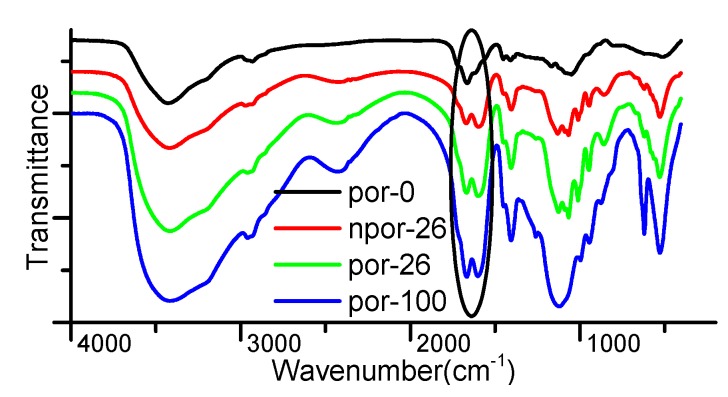
FT-IR spectrum of porous (nonporous) hydrogels soaked in the MnSO_4_ solution with different concentrations as noted in the legend.

FT-IR spectra of the gel samples soaked in MnSO_4_ solution provides additional evidence that Mn^2+^ should be involved in the gel structure. Figure 4 shows that there are acrylic acid-associated structures in the gel (peaks around 1670 cm^−1^). It should be the Mn^2+^ formed complex and physical crosslinks that contribute to the formation of the associated structures, since the peak is strengthened significantly with the increase of MnSO_4_ concentration. In the MnSO_4_ solution with the same concentration, the porous gel has a stronger associated structure absorption than the non-porous gel, which shows from another angle that SA in the porous gel enhanced the binding of Mn^2+^.

### 3.3. Mn^2+^ Binding Alternated Oscillation

**Figure 5:**
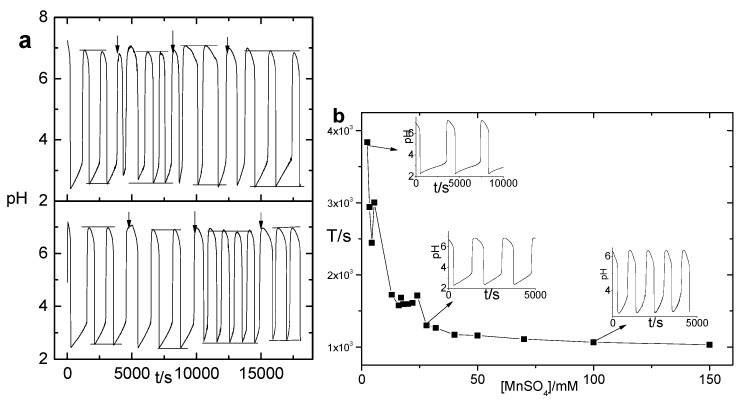
(**a**) pH oscillation sequences of the porous hydrogel-BSM coupled system. The two sequences are realized with identical parameter settings, i.e., both the BSM setting and gel component size are the same. The arrows indicate the time at which a new porous gel sample was added. The sizes of the three gels added in sequence are 10 × 7 × 3, 10 × 5 × 3, and 10 × 3 × 3 mm^3^, respectively. (**b**) Dependence of the BSM oscillation period on the concentration of MnSO_4_, and other experimental parameters, are the same as Figure 1a.

The special coupling mechanism also changes the bulk oscillation character. Figure 5a shows two independent oscillation sequences when porous gels of the same size were put into oscillation with identical parameter settings. The oscillation period is randomly reduced or increased by the addition of the gel. This is also associated with the binding of Mn^2+^. When pH-dependent processes are driven by pH oscillations, they usually coupled through H^+^ and have a buffer-like effect to the oscillation, which will reduce the amplitude as well as the period of the oscillation [45,46]. This is because H^+^ consumption in pH-dependent processes speeds up the H^+^ production in the reversible ionization process in the oscillation. For the BSM system, its amplitude is robust [47], but its period will be reduced. However, coupling through Mn^2+^ has quite the opposite effect. Mn^2+^ consumes H^+^ in BSM, and its binding in the gel will retard the negative feedback of BSM and prolong its period. The experimental results of the BSM system (Figure 5b) also verifies that the BSM period increases with the decreasing of Mn^2+^ concentration. Note that the system described in Figure 5b has no hydrogel in it. Therefore, the irregular period change in Figure 5a should be the result of competition binding of the two coupling media, i.e., H^+^ and Mn^2+^. Since the monomer distribution inside the gel is not homogenous, the oscillation sequence cannot be quantitatively reproducible.

Increasing the gel size or enhancing the negatively-charged gel ingredient content (introducing some 2-acrylamide-2-methylpropanesulfonic acid (AMPS) in the copolymer) may stop the oscillation at high pHs, with a large amount of white precipitate found around the gel. The precipitation should be the insoluble Mn(OH)_2_ formed in the high pH environment when local Mn^2+^ concentration is high. When BSM is conducted at higher MnSO_4_ concentration (above 13 mM), a small amount of white precipitate could be found at the bottom of reactor, indicating the possibility of forming the insoluble Mn(OH)_2_ in BSM reactions. In the current system, most precipitates are formed around the gel, suggesting Mn^2+^ ions are gathered near the gel so that its local concentration is high enough to form a precipitate. The formation of such a large amount of precipitate makes the Mn^2+^ in the solution less than necessary for the oscillation, i.e., it cuts off the feedback loop. Therefore, the oscillation stops at a high pH when the precipitate condition is satisfied.

## 4. Conclusions

Large amplitude hydrogel volume oscillation was observed in a chemomechanical system constituted of a BSM pH oscillatory reaction and porous p(AA-*co*-AM) gel treated with alginate as the pore-forming agent. The negative feedback species Mn^2+^ is involved in the chemomechanical coupling by forming complex and physical crosslinks within the gel. These constitute additional three-dimensional networks in the hydrogel, which considerably reinforced the gel network structure. Despite that the nonlinear stability still originating from the oscillatory reaction, Mn^2+^ binding also helps to maintain the chemomechanical oscillation by strengthening the gel. As oscillatory reactions are currently used as a non-equilibrium driving force to produce autonomous behaviors in many areas, the negative feedback species involved coupling mechanism provide new sight for the related chemomechanical transitions.

## Supplementary Materials

Supplementary materials are available online at www.mdpi.com/2073-4360/9/11/554/s1.
