# Supplementary material for: Experimental Evidence of Large Amplitude pH Mediated Autonomous Chemomechanical Oscillation
Source: Polymers (Basel). 2017 Oct 25;9(11):554. doi: 10.3390/polym9110554 (PMC6418536; doi:10.3390/polym9110554)
Supplement: Supplementary file 1 [file polymers-09-00554-s001.zip › polymers-233591-supplementary.pdf]

Experimental evidence of large amplitude pH mediated autonomous chemomechanical oscillation

Xin Yang [1], Yi Zhou, [1, 2 ǂ] Lin Ji[1,*] Yanhui Ding [1], and Jianquan Wang [2,*] Xin Liang[3]

Supporting Information

Video of the oscillating gel.

Video legend: The video of oscillating gel in the porous P(AA-*co*-AM) hydrogel and BSM pH oscillatory reaction coupled system, with the corresponding quantitative data shown in Fig 1.
